# Do neurooncological patients and their significant others agree on quality of life ratings?

**DOI:** 10.1186/1477-7525-7-87

**Published:** 2009-10-09

**Authors:** Johannes M Giesinger, Miriam Golser, Astrid Erharter, Georg Kemmler, Gabriele Schauer-Maurer, Guenter Stockhammer, Armin Muigg, Markus Hutterer, Gerhard Rumpold, Bernhard Holzner

**Affiliations:** 1 Department of Psychiatry and Psychotherapy, Innsbruck Medical University, Anichstr.35, A-6020 Innsbruck, Austria; 2 Department of Neurology, Innsbruck Medical University, Anichstr. 35, A-6020 Innsbruck, Austria

## Abstract

**Introduction:**

Patients suffering from brain tumours often experience a wide range of cognitive impairments that impair their ability to report on their quality of life and symptom burden. The use of proxy ratings by significant others may be a promising alternative to gain information for medical decision making or research purposes, if self-ratings are not obtainable. Our study investigated the agreement of quality of life and symptom ratings by the patient him/herself or by a significant other.

**Methods:**

Patients with primary brain tumours were recruited at the neurooncological outpatient unit of Innsbruck Medical University. Quality of life self- and proxy-ratings were collected using the EORTC QLQ-C30 and its brain cancer module, the QLQ-BN20.

**Results:**

Between May 2005 and August 2007, 42 pairs consisting of a patient and his/her significant other were included in the study. Most of the employed quality of life scales showed fairly good agreement between patient- and proxy-ratings (median correlation 0.46). This was especially true for Physical Functioning, Sleeping Disturbances, Appetite Loss, Constipation, Taste Alterations, Visual Disorders, Motor Dysfunction, Communication Deficits, Hair Loss, Itchy Skin, Motor Dysfunction and Hair Loss. Worse rater agreement was found for Social Functioning, Emotional Functioning, Cognitive Functioning, Fatigue, Pain, Dyspnoea and Seizures.

**Conclusion:**

The assessment of quality of life in brain cancer patients through ratings from their significant others seems to be a feasible strategy to gain information about certain aspects of patient's quality of life and symptom burden, if the patient is not able to provide information himself.

## Introduction

The assessment of patient-reported outcomes (PRO) has become very common in oncological research and to a lesser degree in daily clinical routine. Information gathered through PRO-monitoring, especially data on quality of life (QOL), has proved to be useful in symptom management and evaluation of oncological treatment [1-5]. But to date the number of studies on QOL in patients with brain tumours is limited, although the limited curative options underline the importance of QOL.

Naturally, the assessment of PRO is restricted to patients having the ability to report on what they experience throughout the course of the disease. In patients with brain tumours the assessment of QOL can prove difficult not only due to physical condition but also because of cognitive impairments such as lack of concentration, thought disorder, communication deficits and visual disorders.

If during the course of the disease the patient's ability to report on his QOL and symptoms diminishes, ratings by others gain importance. Since significant others such as spouses, children or other family members are often intimately involved in patient care, their impression of a patient's well-being could contribute to symptom management and treatment evaluation if gathering information from the patient is not possible. In a research context proxy ratings may reduce drop out bias by allowing patients with progressive cognitive deterioration to remain in the study.

There is some evidence that significant others show agreement with patients' self-ratings on QOL for various types of cancer, although proxies tend to underrate QOL. Furthermore, agreement is lower for psychosocial issues and higher for physical symptoms [6-9].

This kind of proxy-ratings was also found to be more concordant with patients' self-ratings than ratings by physicians [10,11]. Besides neurooncological patients, proxy-ratings have also been proven useful in many other patient groups that can not be assessed directly, e.g. in patients suffering from dementia [12] or in children [13].

Obviously, the usefulness of a proxy-approach to PRO-assessment depends strongly on the reliability of the rating in terms of agreement with the patient's self-rating. Therefore it is of interest whether or not self- and proxy-ratings correlate highly and whether or not there is a bias induced by proxies over- or underestimating patients' QOL.

The current study aimed to investigate the relation between ratings of patients and their significant others on QOL assessed with the EORTC QLQ-C30 and QLQ-BN20. Thus, we addressed the following questions:

1.) To what degree do self- and proxy-ratings on QOL correlate?

2.) Is there a systematic difference between self- and proxy-ratings on QOL?

3.) What percentage of ratings on QOL show strong agreement?

## Methods

### Sample

Patients with primary brain tumors treated at the neurooncological outpatient unit of Innsbruck Medical University were considered for participation in the study. Inclusion criteria were age between 18 and 80 years, fluency in German, no severe cognitive impairments, an expected survival time of at least 3 months and informed consent. As „severe cognitive impairment" we considered a degree of impairment not allowing the patient to report on his QOL. Exclusion criteria were very bad physical condition as rated by the treating physician and visiting the outpatient unit less than once a year. In addition to patients' ratings proxy-ratings from a significant other were collected. Significant others comprised (de facto) spouses, children (aged above 18 years), siblings or any person living with the patient. Informed consent was collected from participating significant others as well. The study was approved by the Ethics Committee of Innsbruck Medical University.

### Procedure

Patients and their significant others were approached while waiting for their examination at the neurooncological outpatient unit. Data collection was done partly by a graduate psychology student and partly by nurses. After providing informed consent tablet-PCs presenting the EORTC QLQ-C30 and QLQ-BN20 on the screen were handed over to the patients and significant others along with instructions for the completion of the questionnaires. They filled in the questionnaires simultaneously and were asked to do so independently. The student or nurse supvervised data entry, escpecially with regard to possible communication between patient and significant other. As software tool for data collection we used a program called Computer-based Health Evaluation System [CHES, [14]]. CHES is a PC-program for the computerised assessment, calculation and presentation of psychosocial and medical data.

### Assessment Instruments

#### EORTC QLQ-C30

The EORTC QLQ-C30 [15], an internationally validated and widely used cancer-specific QOL-instrument, assesses various facets of functioning, symptoms common in cancer patients and global QOL. The EORTC quality of life questionnaire suite has a modular structure consisting of a core questionnaire (EORTC QLQ-C30) and specific additional modules for cancer patients of different diagnostic groups. As a supplement two items concerning taste and smell alteration were added from the EORTC Quality of Life Group item bank ("Have you had problems with your sense of taste?" and "Did food and drink taste different from usual?"). This item bank covers all items included in the QLQ-C30 and its various modules. The two items on taste were summed to generate a novel subscale called the Taste Alterations subscale.

For collection of proxy-ratings the items were altered to refer to the patient in the third person, instead of the first person self-rating version.

#### EORTC QLQ-BN20

The Brain Cancer Module (EORTC QLQ-BN20 [16]) is a 20-item supplement for the QLQ-C30 to assess brain cancer-specific QOL issues. The module comprises the subscales Future Uncertainty, Visual Disorders, Bladder control, Motor Dysfunctions, Headaches, Communication Deficits, Seizures, Hair Loss, Itchy Skin and Weakness of Legs.

Again the wording of the items was altered to third person for proxy-ratings.

### Statistical analysis

Patient and significant other scores on the QLQ-C30 and QLQ-BN20 were summarised as means and standard deviations. All scales were scored according to the EORTC guidelines along a possible range from 0 to 100 points.

T-tests for dependent samples were used to detect any systematic differences, while correlations between self- and proxy-ratings were carried out using the Pearson-correlation coefficient. 95%-confidence intervals were calculated for all correlation coefficients. Since correlations only reflect the strength of relation between ratings, but do not reflect systematic differences, the T-tests appeared to be more meaningful in determining rater agreement. Following recommendations of Osoba et al. [17] and King [18] we considered mean differences between patient and proxy ratings equal or below 5 points as an indicator of good rater agreement.

As an additional measure of agreement between patients and significant others we calculated the percentage of ratings with differences ≤5 points for each scale.

To demonstrate the extent of rater disagreement across the range of a scale we provide Bland and Altman plots [19].

Power analysis was done for detecting mean differences between patient and proxy ratings. A sample of 42 patient-proxy-pairs was found to be sufficient to detect a mean difference with an effect size of 0.44 (two-sided test, power = 0.80, alpha = 0.05).

## Results

### Sample characteristics

Between May 2005 and August 2007, 157 patients with primary brain tumors treated at the neurooncological outpatient unit of Innsbruck Medical University were eligible for participation in the study. The included patients were a sub-sample of a larger study on patient-reported outcome monitoring in neurooncologial patients. More details on data collection can be found in Erharter et al [20].

A total of 47 patients could not be included (19 patients were in very bad physical condition, 18 patients visited the outpatient unit less frequently than once per year, 4 patients did not provide informed consent, 3 patients were not fluent in German and 3 patients had severe visual disorders). Thus, data from 110 patients were available. Additional ratings from significant others could be collected for 42 patients (43 significant others refused participation, 25 patients did not bring a significant other with them), i.e. 42 paired ratings were available for statistical analysis. Details on sociodemographic and clinical variables are shown in Table 1.

**Table 1:** Descriptive statistics for sociodemographic and clinical data at baseline (N = 42)

**Age (years)**	**Mean (SD)**	**47.5 (14.2)**
Sex	Women	47%
	Men	52%

Marital status	Single	12%
	Partnership, marriage	81%
	Divorced, seperated	2%
	Widowed	5%

Housing situation	living alone	3%
	living in partnership/with children and/or with children	86%
	living with family of origin	7%
	nursing home	3%

Education	Compulsory school	24%
	Apprenticeship, professional school	41%
	A-level	29%
	University degree	6%

Employment status	Full time	31%
	Part time	8%
	Homemaker	19%
	Training	3%
	Retired	19%
	Status of employee's illness	6%
	Others	14%

Significant Other	Spouse	73%
	Child	18%
	Parent	6% 1%
	Friend	3%

Duration of illness (months)	Mean (SD)	49.3 (47.8)

Tumor type	Meningioma	5%
	Glioblastoma	16%
	Astrocytoma	41%
	Oligodendroglioma	24%
	Ependymoma	3%
	Other	11%

WHO-Grading	Grade I	0.0%
	Grade II	45%
	Grade III	35%
	Grade IV	21%

Previous surgery	no surgery/biopsy	46%
	Partial resection	29%
	Total resection	25%

Previous radiotherapy		63%

Previous chemotherapy		53%

Location of tumor	Right hemisphere	57%
	Left hemisphere	43%

### Agreement between self-ratings and proxy-ratings for the QLQ-C30

For 14 of the 16 subscales (including the Taste Alterations subscale) differences between patients' self-ratings and proxy-ratings by a significant other were below 5 points. Higher discrepancies were only found for Social Functioning (patient mean 8.7 points higher than proxy-mean) and Dyspnoea (patient mean 5.6 points higher than proxy-mean). Seven of the 16 subscales showed correlations between self- and proxy-ratings of at least 0.5. Coefficients were highest for Physical Functioning (r = 0.79) and Taste Alterations (r = 0.77) and lowest for Social Functioning (r = 0.26, not significant) and Pain (r = 0.28, not significant).

**Figure 1:**
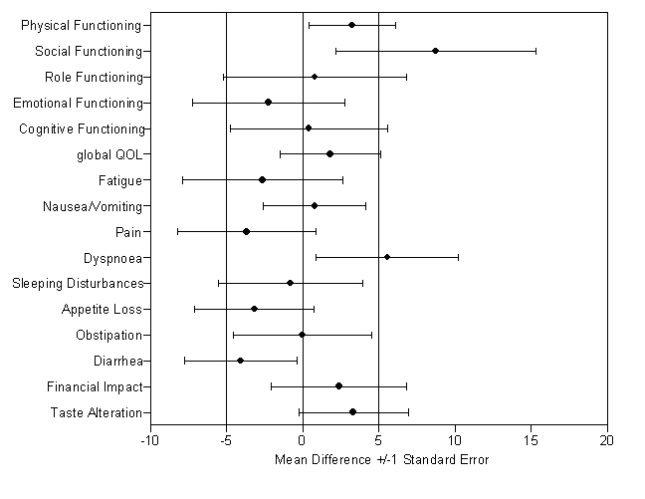
**Mean Differences (Patients minus Proxy) for the QLQ-C30 (dashed reference lines indicate margin for a relevant difference)**.

**Figure 2:**
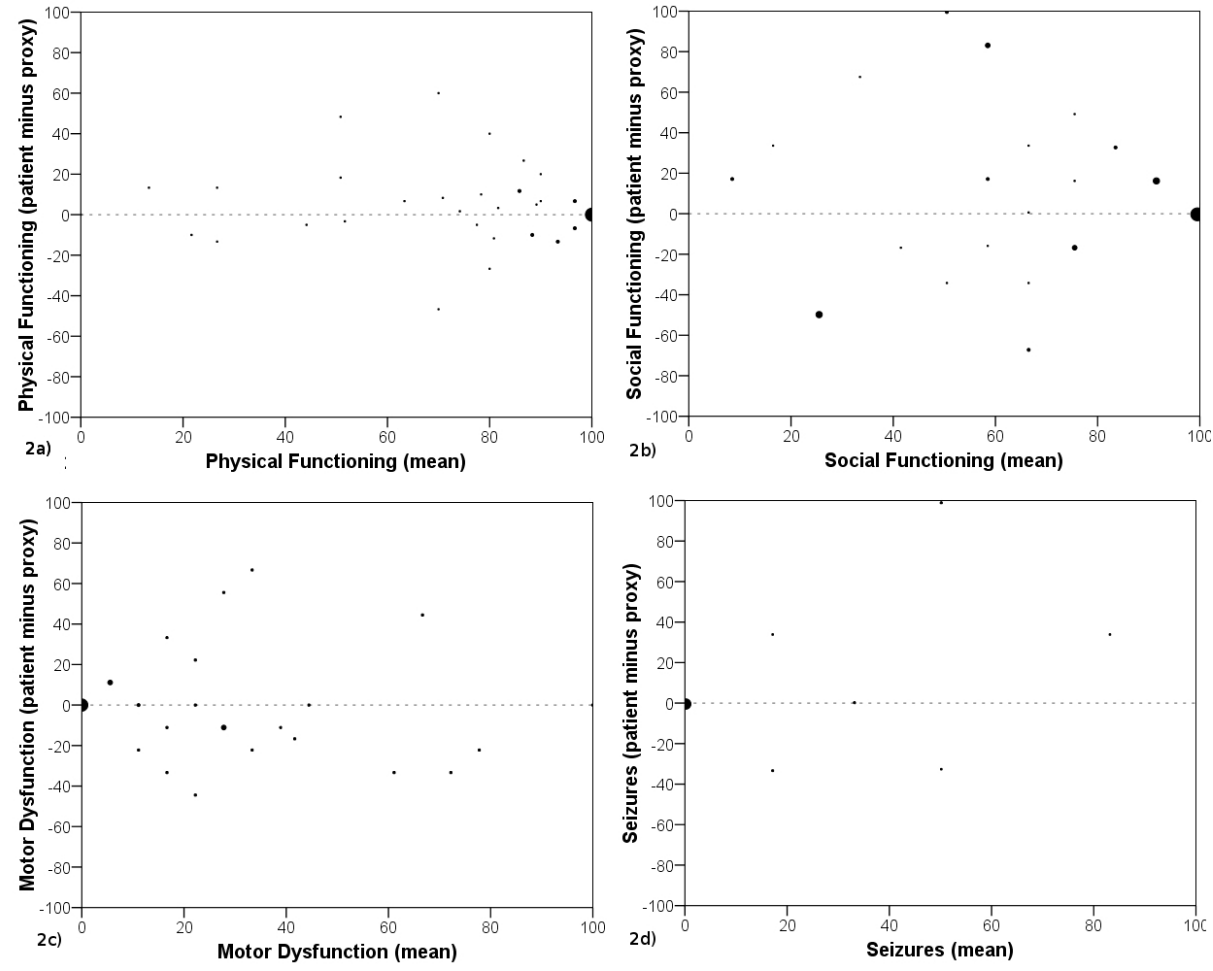
**Bland and Altman plots for Physical Functioning (2a), Social Functioning (2b), Motor Dysfunction (2c) and Seizures (2d)**.

Accuracy, in terms of percentage of differences equal or below 5 points, was highest for Diarrhea (83%), Appetite Loss (71%) and Constipation (68%) and lowest for Emotional Functioning (14%), Fatigue (19%) and Social Functioning (21%). For 8 of the 16 scales the percentage of differences equal or below 5 points was at least 50%. For further details see Table 2 and Figure 1. To illustrate extent of rater agreement across the scale range Bland and Altman plots are shown for Physical Functioning (Figure 2a) and Social Functioning (Figure 2b).

**Table 2:** Agreement of patient- and proxy-ratings for the EORTC QLQ-C30

**EORTC QLQ-C30**	**Patient Mean (SD)**	**Proxy Mean (SD)**	**Patient minus Proxy**	**effect size**	**t-value** **p-value**	**Pearson-Correlation** **(CI95%)**	**agreement (+/- 5 points)**
Physical Functioning	77.6 (27.3)	74.3 (28.8)	3.3	0.12	t = 1.16; p = 0.25	0.79* (0.65-0.89)	36%
Social Functioning	69.8 (35.4)	61.1 (34.5)	8.7	0.25	t = 1.33; p = 0.19	0.26 (-0.05-0.53)	21%
Role Functioning	63.5 (36.9)	62.7 (35.3)	0.8	0.02	t = 0.13; p = 0.90	0.42* (0.13-0.65)	31%
Emotional Functioning	59.5 (30.4)	61.8 (23.8)	-2.3	-0.08	t = -0.45; p = 0.65	0.31* (0.01-0.56)	14%
Cognitive Functioning	70.6 (31.2)	70.2 (27.7)	0.4	0.01	t = 0.08; p = 0.94	0.36* (0.06-0.60)	24%
Global QOL	63.8 (23.0)	62.0 (21.6)	1.8	0.08	t = 0.55; p = 0.58	0.55* (0.29-0.74)	24%

Fatigue	41.5 (32.6)	44.2 (29.3)	-2.7	-0.09	t = -0.50; p = 0.62	0.40* (0.11-0.64)	19%
Nausea/Vomiting	9.9 (16.9)	9.1 (20.9)	0.8	0.04	t = 0.24; p = 0.81	0.35* (0.05-0.60)	60%
Pain	15.9 (25.5)	19.5 (22.6)	-3.7	-0.14	t = -0.81; p = 0.42	0.28 (-0.03-0.54)	39%
Dyspnoea	20.6 (31.2)	15.1 (22.3)	5.6	0.20	t = 1.19; p = 0.24	0.40* (0.11-0.64)	50%
Sleeping Disturbances	27.8 (32.0)	28.6 (30.0)	-0.8	-0.03	t = -0.17; p = 0.87	0.51* (0.25-0.71)	52%
Appetite Loss	15.9 (27.8)	19.0 (29.6)	-3.2	-0.11	t = -0.81; p = 0.42	0.61* (0.38-0.78)	71%
Constipation	15.8 (29.8)	15.8 (25.4)	0.0	0.00	t = 0.00; p = 1.00	0.50* (0.24-0.70)	68%
Diarrhea	7.3 (19.0)	11.4 (25.4)	-4.1	-0.17	t = -1.09; p = 0.28	0.46* (0.18-0.67)	83%
Financial Impact	22.2 (31.8)	19.8 (27.6)	2.4	0.08	t = 0.53; p = 0.60	0.53* (0.28-0.72)	60%

Taste Alterations	22.1 (34.7)	18.8 (32.5)	3.3	0.10	t = 0.93; p = 0.36	0.77* (0.62-0.88)	60%

*p < 0.05

### Agreement between self-ratings and proxy-ratings for the QLQ-BN20

For 10 of the 11 scales of the brain tumour module mean differences between patients' self-ratings and proxy-ratings by a significant other were below 5 points. A higher discrepancy was only found for Seizures (patient mean 6.3 points higher than proxy mean).

Correlations between self- and proxy-ratings were at least 0.5 for 6 of the 11 scales. Coefficients were highest for Motor Dysfunction (r = 0.67) and Communication Deficits (r = 0.67) and lowest for Bladder Control (r = 0.14) and Seizures (r = 0.38).

**Figure 3:**
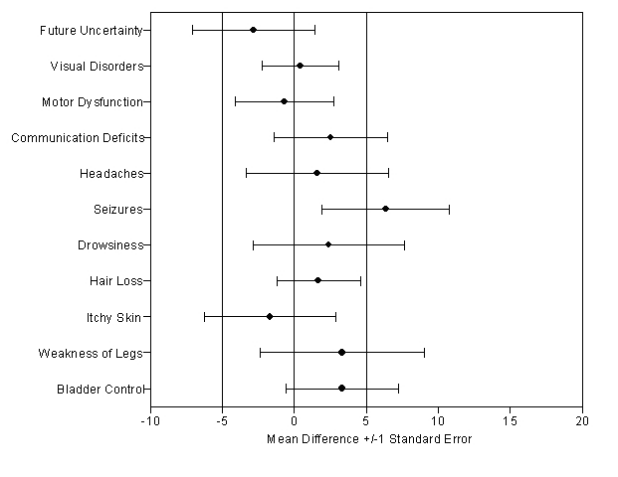
**Mean Differences (Patients minus Proxy) for the QLQ-BN20 (dashed reference lines indicate margin for a relevant difference)**.

Accuracy, in terms of percentage of differences equal or below 5 points, was highest for Seizures (81%), Hair Loss (78%) and Bladder Control (75%) and lowest for Future Uncertainty (29%), Drowsiness (38%) and Motor Dysfunction (44%). For 7 of the 11 scales the percentage of differences equal or below 5 points was at least 50%. For further details see Table 3 and Figure 3.

**Table 3:** Agreement of patient- and proxy-ratings for the EORTC QLQ-BN20

**EORTC QLQ-BN20**	**Patient Mean (SD)**	**Proxy Mean (SD)**	**Patient minus Proxy**	**effect size**	**t-value** **p-value**	**Pearson-Correlation** **(CI95%)**	**agreement (+/- 5 point)**
Future Uncertainty	28.3 (29.6)	31.1 (28.1)	-2.8	-0.10	t = -0.67; p = 0.51	0.55* (0.29-0.74)	29%
Visual Disorders	13.3 (16.5)	12.9 (19.9)	0.4	0.02	t = 0.16; p = 0.88	0.58* (0.34-0.76)	50%
Motor Dysfunction	21.1 (25.9)	21.8 (28.3)	-0.7	-0.02	t = -0.20; p = 0.84	0.67* (0.46-0.81)	44%
Communication Deficits	26.3 (28.1)	23.8 (33.4)	2.5	0.08	t = 0.64; p = 0.53	0.67* (0.46-0.81)	45%
Headaches	34.1 (35.7)	32.5 (34.9)	1.6	0.04	t = 0.32; p = 0.75	0.59* (0.35-0.77)	57%
Seizures	13.5 (30.4)	7.1 (17.3)	6.3	0.27	t = 1.43; p = 0.16	0.38* (0.09-0.62)	81%
Drowsiness	38.9 (32.9)	36.5 (30.2)	2.4	0.08	t = 0.45; p = 0.65	0.42* (0.13-0.65)	38%
Hair Loss	9.2 (18.5)	7.5 (19.2)	1.7	0.09	t = 0.57; p = 0.57	0.52* (0.26-0.72)	78%
Itchy Skin	12.8 (22.4)	14.5 (29.4)	-1.7	-0.07	t = -0.37; p = 0.71	0.42* (0.13-0.65)	64%
Weakness of Legs	25.0 (36.8)	21.7 (31.6)	3.3	0.10	t = 0.58; p = 0.56	0.45* (0.17-0.66)	60%
Bladder Control	10.0 (21.6)	6.7 (15.5)	3.3	0.18	t = 0.85; p = 0.40	0.14 (-0.17-0.43)	75%

*p < 0.05

Bland and Altman plots are shown for Motor Dysfunction (Figure 2c) and Seizures (Figure 2d) to exemplify extent of rater agreement across the scale range.

## Discussion

The comparison of patients' rating on their QOL with proxy-ratings obtained from their significant others is of importance to the decision whether or not these proxy-ratings are a useful measure, if patients' ability to report on his QOL diminishes due to physical or cognitive deterioration.

Our study found that for a considerable number of subscales of the EORTC QLQ-C30 and QLQ-BN20 proxy-ratings by significant others can be regarded as useful. This was especially true for Physical Functioning, Sleeping Disturbances, Appetite Loss, Constipation, Financial Impact and Taste Alterations. Worse rater agreement was found for Social Functioning, Emotional Functioning, Cognitive Functioning, Fatigue, Pain, Dyspnoea and Seizures. For these scales correlations as well as percentage of agreement (+/-5 points) were low. However, with the exception of Social Functioning and Dyspnoea means of patients' ratings and proxy-ratings were rather similar (less than 5 points difference).

The additional module QLQ-BN20 showed fairly good rater agreement for most scales. Worst agreement was found for Seizures and Bladder Control.

With reference to Osoba et al. [17] and King [18] we considered mean differences above 5 points as relevant rater disagreement. Taking this into account discrepancies between proxy- and self-ratings were rather insiginficant for most scales. No uniform pattern was found with respect to systematic under/over-rating by proxies.

Another important issue is the extent of rater-agreement across the scale range, especially with regard to generalisability of our results to patients in a poor condition. Analysis of Bland and Altman plots indicate that agreement is worst for the central section of a scale. This finding is probably a result of the fact that possible differences between raters are necessarily minimised by the limited range scale.

Overall, proxy-ratings performed somewhat better for more overt aspects of QOL such as physical symptoms, whereas ratings on social and psychological aspects showed less congruency.

A limitation of our study is the small sample size which did not allow to detect small mean differences between patient and proxy ratings. For the same reason, it was not possible to perform subgroup analyses on certain patient groups. In addition, patients in a very bad physical condition, would have been of importance to our study, as proxy-ratings are most useful in that patient group. However, due to ethical considerations it was not possible to include such, since burden caused by filling in both questionnaires was considered not acceptable for these patients. Another limitation of our study is the high rate of significant others refusing participation in the study.

The results for accuracy (percentage of mean differences equal or below 5 points) may have been affected by the number of items in a scale, more precisely the number of possible scores on a scale. Two contrary effects can be expected from this. On the one hand a low number of possible scores increases agreement due to chance, on the other hand if the distance between two possible scores is higher than 10 points (e.g. for scales containing one or two items) only exact agreement is taken into account by this accuracy parameter.

The study most similar to ours [6] found more pronounced mean differences for Physical Functioning, Role Functioning, Cognitive Functioning, Social Functioning and Fatigue (all between 5 and 10 points). With the exception of Physical Functioning, these scales showed also only a moderate proportion of exact agreement. A slight difference to our study was the use of a previous version of the QLQ-C30 in the study by Sneeuw et al. [6] that employed a dichotomous response format for the scales Physical Functioning and Role Functioning.

Proxies' relationship with the patient, age, gender and culture showed no significant association with rater agreement. But agreement was worse in patients with mental confusion, cognitive impairments and motor deficits. We think that the finding that rater agreement is low in patients with severe cognitive impairments should not be considered per se as an indication for inaccurate proxy rating. It might also reflect patients' inability to report on their condition. On the other hand, it may as well be difficult for proxies to understand the individual consequences of cognitive decline. Additional clinical variables as more objective criteria may be helpful in evaluating rater disagreement in this patient group.

In a recent study by Brown et al. [21] on rater agreement in patients with newly diagnosed high-grade gliomas proxy-ratings by a caregiver chosen by the patient himself also showed good congruence. As QOL-instrument this study employed the FACT-Br [22]. Correlation between patient-ratings and caregiver-ratings was 0.63 at baseline and 0.64 at 2 and 4 months follow-up, percentage of agreement (+/- 10 points on a scale ranging from 0 to 100) was 63-68% at the three assessment time points.

With regard to type of proxy-rating, proxy-raters can not only differ in their relation to the patient (significant other, treating physician, caregiver etc.) but also in the perspective they take towards the patient. Gundy and Aaronson [23] investigated whether or not there are differences in proxy-ratings if the proxy rates the patient taking the patient's perspective or if he makes his own assessment of the patient. No differences with regard to bias were found between both types of ratings, although it should be mentioned that the study might have been not sufficiently powered to detect possible differences between these types of ratings.

Taking our own findings and those from similar studies into account, the assessment of QOL in brain cancer patients through ratings from their significant others seems to be a feasible strategy to gain information about important aspects of a patient's QOL, if the patient is not able to provide information himself. However, in general rater agreement is lower for psychosocial issues compared to physical symptoms.

In a research context proxy ratings may allow to reduce bias from patients droping out of studies because of deteriorating health and in a clinical context proxy-ratings could contribute to medical decision making. Future research, should further evaluate the impact of patient and proxy characteristics on rater agreement and include further criteria for accuracy of proxy ratings.

## List of abbreviations

CHES: Computer-based Health Evaluation System; CI95%: 95% confidence interval; EORTC: European Organisation for Research and Treatment of Cancer; FACT-Br: Functional Assessment of Cancer Therapy - Brain; PRO: Patient-reported Outcome; QLQ-BN20: Quality of Life Questionnaire - Brain Cancer Module; QLQ-C30: Quality of Life Questionnaire - Core 30; QOL: Quality of Life; SD: Standard deviation; WHO: World Health Organisation;

## Competing interests

The authors declare that they have no competing interests.

## Authors' contributions

GJ, GM, EA and HB were responsible for study design, conceptualization and writing of the manuscript as well as for data collection. MA, HM and SG were the treating neurologists and therefore in charge of patient recruitment and gave important input for medical content. GJ and KG performed the statistical analysis. RG and SMG helped to draft the manuscript. All authors read and approved the final manuscript.
